# The Regulatory Effects of Lateral Hypothalamus Area GABA_B_ Receptor on Gastric Ischemia-Reperfusion Injury in Rats

**DOI:** 10.3389/fphys.2017.00722

**Published:** 2017-09-21

**Authors:** Lin Gao, Huiru Zhao, Tao Zhu, Yeliu Liu, Li Hu, Zhenguo Liu, Hai Huang, Fuxue Chen, Zhenxu Deng, Dechang Chu, Dongshu Du

**Affiliations:** ^1^Neurology Center, The First Affiliated Hospital of Zhengzhou University Zhengzhou, China; ^2^Shanghai Key Laboratory of Bio-Crops, College of Life Science, Shanghai University Shanghai, China; ^3^Department of Life Science, Heze University Heze, China; ^4^Department of General Surgery, Huai'an First People's Hospital, Nanjing Medical University Huai'an, China

**Keywords:** lateral hypothalamic area (LHA), GABA_B_ receptor, gastric ischemia-reperfusion injury (GI-RI), inflammatory factor, oxidative stress

## Abstract

**HIGHLIGHTS**

- The aim of the research was to determine the functional effects and molecular mechanisms of GABA_B_ receptor on ischemia reperfusion-induced gastric injury in rats.
- The lateral hypothalamus area GABA_B_ receptor attenuated the ischemia reperfusion-induced gastric injury by up-regulating the production of GABA, GABA_B_R, and down-regulating *P-*GABA_B_R in the brain.
- This work would provide a new therapeutic strategy for acute gastric injury.

Gastric ischemia-reperfusion (GI-R) injury progression is largely associated with excessive activation of the greater splanchnic nerve (GSN). This study aims to investigate the protective effects of GABA_B_ receptor (GABA_B_R) in the lateral hypothalamic area (LHA) on GI-R injury. A model of GI-R injury was established by clamping the celiac artery for 30 min and then reperfusion for 1 h. The coordinate of FN and LHA was identified in Stereotaxic Coordinates and then the *L-*Glu was microinjected into FN, GABA_B_ receptor agonist baclofen, or GABA_B_ receptor antagonist CGP35348 was microinjected into the LHA, finally the GI-R model was prepared. The expression of GABA_B_R, *P*-GABA_B_R, NOX2, NOX4, and SOD in the LHA was detected by western blot, PCR, and RT-PCR. The expression of IL-1β, NOX2, and NXO4 in gastric mucosa was detected by western blot. We found that microinjection of *L-*Glu into the FN or GABA_B_ receptor agonist (baclofen) into the LHA attenuated GI-R injury. Pretreatment with GABA_B_ receptor antagonist CGP35348 reversed the protective effects of FN stimulation or baclofen into the LHA. Microinjection of baclofen into the LHA obviously reduced the expression of inflammatory factor IL-1β, NOX2, and NOX4 in the gastric mucosa.

**Conclusion:** The protective effects of microinjection of GABA_B_R agonist into LHA on GI-R injury in rats could be mediated by up-regulating the production of GABA, GABA_B_R, and down-regulating *P*-GABA_B_R in the LHA.

## Introduction

Many surgery-evoked ischemia have been suggested to induce gastric mucosal injury as well as gastrointestinal dysmotility (Gross and Auchampach, 2007; Tsukamoto et al., 2011). The fastigial nucleus (FN), hypothalamic paraventricular nucleus (PVN) (Zhang et al., 2002, 2007), and the lateral hypothalamic area (LHA) (Zhu J. Z. et al., 2012) are three major nuclei that can regulate gastric activity and gastric mucosal injury in GI-R model.

The amino acid γ-aminobutyric acid (GABA), a major inhibitory neurotransmitter of the central nervous system (CNS), was involved in regulating multiple physiology activities including sedation and anxiolytic, muscle relaxation, analgesia, and anticonvulsant activities (Minocha and Galligan, 1993; Enna and McCarson, 2006; Page et al., 2006). The previous study demonstrated that the GABA receptors were expressed on the plasma membrane of neurons in LHA (Sieghart and Sperk, 2002).

The GABA receptors were divided into three receptor complexes containing the ionotropic GABA_A/C_ or the metabotropic GABA_B_ receptor (Page and Blackshaw, 1999). Our previous studies showed that chemical stimulation of FN with *L-*Glu attenuated gastric ischemia- reperfusion injury, microinjection of GABA_A_ receptor antagonist into LHA aggravated GI-R injury (Du et al., 2013), cerebellar-hypothalamic circuits modulate the gastric mucosal injury induced by ischemia-reperfusion. Furthermore, a recent study indicated that GABA_A_ receptor overexpression in the LHA attenuated gastric ischemia-reperfusion injury in rats (Gao et al., 2015).

However, little is known about the effects of GABA_B_ receptor in the LHA on GI-R injury. Based on our previous reports (Du et al., 2013; Gao et al., 2015), we calculate GABA_B_ receptor in the LHA could alleviate the GI-R injury. The nicotinamide adenine dinucleotide phosphate-oxidase (NOX) is a membrane-bound enzyme complex, which obviously induces tissues ischemia injury in the central and peripheral tissues (Chen et al., 2013; Liu et al., 2017). IL-1β also known as leukocytic pyrogen, mononuclear cell factor, is a cytokine protein that in human is encoded by the IL1B genen. Furthermore, our previous report demonstrated that IL-1β was involved in the regulation of myocardium ischemia injury (Du et al., 2015). Therefore, we speculate central GABA_B_ receptor may be coupled with NOX or inflammatory cytokines (IL-1β) in GI-R injury.

Baclofen, a prototypical GABA_B_ receptor agonist, is the most thoroughly study as the widespread use of muscle relaxant drug and adjuvant in pain relief regiment (Patel et al., 2001; van Rijn et al., 2009). Additionally, baclofen has been used as a possible treatment strategy for reflux disease via inhibiting vagally-mediated transient lower esophageal relaxations (Koek et al., 2003). However, the therapeutic potential and molecular mechanism of baclofen on GI-R were unclear. The recent research suggested that baclofen reduced visceromotor and cardiovascular reflexes in rats in response to colorectal distention (Brusberg et al., 2009; Liu et al., 2011). In addition, other study demonstrated that GABA_B_ receptor could induce vasorelaxation in diabetic rat vessels (Kharazmi et al., 2015). However, the effects and mechanism of the GABA_B_ receptor in LHA on GI-R injury are unknown.

Calcitonin gene-related peptide (CGRP) is produced in both peripheral and central neurons and is a peptide vasodilator. In the vascular system, the cell bodies on the trigeminal ganglion are the main source of CGRP, which is thought to play a role in cardiovascular homeostasis. Furthermore, our previous study demonstrated CGRP could reduce the GI-R injury (Du et al., 2010). Therefore, in the present research, we want to confirm the protective effects of GABA_B_ receptor in the LHA be mediated by CGRP neuron derived from dorsal root ganglion, that release CGRP into gastric mucosa.

The specific aim of this study is to investigate the effects of GABA_B_ receptor on GI-R injury. In order to test the potential molecular mechanism of GABA_B_ receptor in the progression of GI-R injury, the GBAB_B_ receptor antagonist CGP35348 or the GBAB_B_ receptor agonist baclofen was respectively microinjected into the LHA.

## Materials and methods

### Animals

Sprague-Dawley rats (adult male), weighting 200–220 g were used (SLRC LABORATORY ANIMAL, Shanghai, China). All procedures were approved by the Experimental Animal Care and Use Committee of Shanghai University (No. 201506-766). Rats were housed at animal facility with controlled temperature (22–24°C) and photoperiod (12 h light/12 h dark) with freely access to water and food. Before experiments, animals were fasted for 24 h.

### Antibodies and reagents

GABA_B_R1(D-2) (sc-16608), NOX4 (H-300) (sc-30141), and CGRP (sc-8856) were all purchased from Santa Cruz Biotechnology (Santa Cruz Biotechnology, Santa Cruz, CA,USA). Phospho-Ser923 GABA_B_ R1 antibody was from ABGENT (WuXiAppTec, Shanghai, China). Anti-NOX2/gp91phox antibody [EPR6991] (ab129068) was from abcam (San Franciso, CA, USA). Goat anti-mouse IgG (H+L) Cy3 was from abcam (San Franciso, CA, USA). Anti-IL-1 beta antibody ab9722 was from abcam (San Franciso, CA, USA). Superoxide dismutase (SOD) assay lists was from Nanjing Jiancheng Biological Engineering Research Institute (Nanjing, China).

### Chemical stimulation of the FN

Chloral hydrate (450 mg/Kg) was used to anesthetize rats before experiment, and anesthetized rats then were fixed onto a stereotaxic apparatus. The coordinate for stimulating the FN (Paxinos et al., 2009) is AP 11.6 mm, LR 1.0 mm, and H 5.6 mm. *L*-Glu (3 μg in a volume of 0.5 μL saline) was microinjected 15 min before GI-R via a channula connected to a microsyringe with a polyethylene tube. The microinjection was performed for 3 min, and the injection cannula was left *in situ* for another 10 min to prevent backflow.

### Microinjection of baclofen or CGP35348 into LHA

The location of the LHA was determined according to The Rat Brain in Sereotaxic Coordinates (Paxinos et al., 2009): LHA, anteroposterior (AP) 2.0 mm, left-right (LR) 1.5 mm, height (H) 8.3–8.4 mm. Baclofen (0.5, 1.5, 3.0 μg) in a volume of 0.5 μL saline and CGP35348 1.125 μg in a volume of 0.5 μL saline, were microinjected into the LHA via a cannula connected to a microsyringe with a polyethylene tube. The microinjection was performed for 3 min, and the injection cannula was left *in situ* for another 10 min to prevent backflow. Baclofen and CGP35348 were microinjected into the LHA 15 min before GI-R respectively.

### Gastric ischemia-reperfusion injury

GI-R was performed following *L*-Glu, Baclofen or CGP35348 microinjection as previously reported (Du et al., 2013). Briefly, the abdominal cavity was cut open for celiac artery and its adjacent tissues isolation. To induce ischemia, the celiac artery was clamped with a small vascular clamp for 30 min. The clamp was then removed to allow reperfusion for 30 min, 1, 2, 4, 8, 16 h. The rats were euthanized under deep anesthesia after each experiment. The rats' stomachs and brains were removed rapidly. The stomachs were opened along the greater curvature, and the gastric mucosa was grossly examined carefully for ulcers. Part of the brains were stored at −80°C for Western blotting, PCR and real-time PCR analysis. The other part brains were immersed in 4% paraformaldehyde for 48 h, and then immersed in 20% sucrose solution for 1 day, at last immersed in 30% for 1 day, sliced to 30 μm thick, mounted on glass slides for the immunofluorescent assay and ROS test.

### Assessment of the gastric mucosal index

The gastric mucosal injury was assessed as described previously (Zhang et al., 2002) with modification. Briefly, the stomach was spread out, and the gastric mucosal injury index (GMII) was counted. This index is based on a cumulative-length scale: an individual lesion limited to within the mucosal epithelium (including pinpoint erosions, ulcers, and hemorrhagic spots) is scored according to its length, for example, (1), value ≤1 mm; (2), value between 1 and 2 mm; (3), value between 2 and 3 mm. Additionally, For lesions >1 mm wide, the lesion score was doubled. Gastric mucosal injury index is thus the sum of the scores of all lesions, the index was determined by an investigator who was blind to the treatments to avoid researcher bias.

### Immunofluorescence

Immunofluorescence staining was performed as described previously (Zhang et al., 2011; Yang et al., 2012; Xu et al., 2013). The histological sections were localized in the LHA. Brain sections were permeabilized for 30 min with 0.1% Trion-X100 in PBS, then washed with PBS, then brain sections were incubated with primary antibody against GABA_B_R1 (1:200, Santa Cruz Biotechnology) for 2 h at room temperature. After four times washes with PBS, brain sections were incubated with secondary antibodies coupled to Cy3 (1:500, Abcam Inc) for 1 h at room temperature. After washed with PBS, then incubated with 1% DAPI in PBS for 5 min. After four washes with PBS, brain sections were analyzed by a Nikon SA Microphot microscope.

### Western blot analysis

Total protein from LHA and gastric mucosa tissues was extracted and protein concentration in cell lysates was determined using a BCA assay kit (Beyotime Shanghai China). Sixty micrograms of protein, obtained from each sample, were separated using SDS-PAGE (10% gel). Samples were then transferred to polyvinylidene fluoride membrane (Immobulon-P 0.45 μm, Millipore Germany). Five percentage of skim milk was used to block the membranes, which were then incubated overnight at 4°C with the primary antibodies GABA_B_R1 (1:300); CGRP (1:200); NOX4 (1:200); Phospho-Ser923 GABA_B_R1 Antibody (1:300); Anti-NOX2/gp91phox antibody (1:300); β-actin (1:1,000, rabbit, Santa Cruz biotechnology); Anti-IL-1beta antibody ab9722 (1:500). After washed with PBST, secondary antibodies (1:10,000, horseradish peroxidase-linked anti-rabbit or anti-goat, Santa Cruz biotechnology) was applied to the membranes for 1 h at room temperature. ECL (Immobulon, Millipore Germany) and Image Develop were used to visualize immunoblots.

### PCR analysis

TRIzol reagent (Takara technology) was used to extract the total RNA from the LHA nuclei issue. Purification and reverse transcription RNA was performed as follow: first-strand cDNA was synthesized from 3 μg total RNA using TransScript One-Step gDNA Removal and cDNA Synthesis SuperMix (TransGen Biotech). The sequences of GABA_B_R primer used and the predicted amplification sizes are as follows: forward: CATCAACTTCCTGCCTGTG, reverse: GTGTCCATATCCGTCCAG, 247 bp.

### Real time-PCR analysis

Purification and reverse transcription RNA was performed as described above. Real time PCR was performed in a total volume of 50 μL with 1 μL reverse transcribed cDNA, 3 μL of each primer in hot start buffer, 0.5 μL hot start Taq DNA polymerase (Takara technology), 5 μL 10×LA TaqBuffer (Mg^2+^ Plus), and 8 μL dNTP Mixture. The sequences of GABA_B_R and β-Actin primers used and the amplification sizes are as follows, GABA_B_R forward: GGAAGGTGGCATCAGGTA, reverse: CATAGTCCAC AGGCAGGAA, 115 bp; β-Actin forward: GTACCCCATTGAAC ACGG, reverse: TGTGGTGCCAAATCTTCTC, 80 bp.

### Measurement of ROS generation in LHA

The LHA tissue was harvested from the rat brain and washed with ice-cold PBS. Subsequently, the LHA tissue was homogenized using western blot lysis buffer at 0–4°C, following which the LHA homogenate was centrifuged at 2,500 × g for 30 min. The supernatants were harvested and stored at −80°C until SOD activity assays was performed. The protein concentrations were determined by the BCA assay kit (Beyotime Shanghai China). A Xanthine/Xanthine Oxidase method was used to determine SOD activity, by measuring the absorbance value at a wavelength of 450 nm. A SOD Assay kit was used to measure SOD activity, according to the manufacturer's protocol (Nanjing Jiancheng Bioengineering Co., Ltd., Nanjing, China). Total SOD activities were expressed in uints/mg of protein.

The reactive oxygen species were detected on frozen section by ROS Fluorescent Probe-DHE (Vigorous Biotechnology, Beijing, China) recommended dilution of 1:100 (w/v) in 1% PBST for 1 h at room temperature and observed by fluorescence microscopy.

### Statistical analysis

Data were analyzed using one-way ANOVA and *LSD*-test. Prism5 software was used (GraphPad Software, San Diego, CA, USA) to determine statistical difference between groups and statistical significance was accepted as *P* < 0.05. Data are presented as mean ± *SD*.

## Results

### Effects of different doses of baclofen and different reperfusion time points on gastric injury

**Figure 1:**
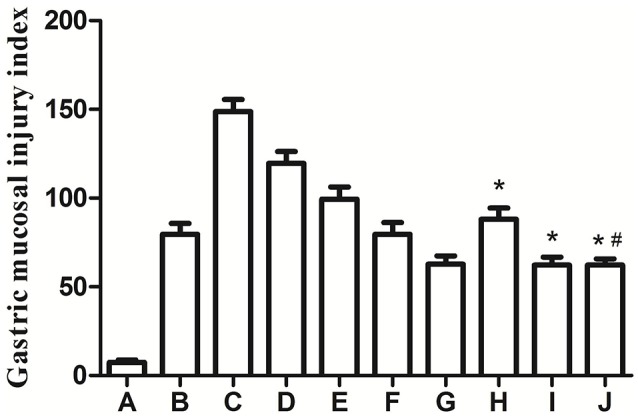
Effects of different doses of Baclofen and different reperfusion time points on gastric injury. ^*^P < 0.001, as compared with the GI-R (1 h) group (C), ^#^*P* > 0.05, as compared with the LHA+GI-R+Baclofen group (0.5 μg, H). A, Normal group; B, GI-R group; C, GI-R (1 h) group; D, GI-R (2 h) group; E, GI-R (4 h) group; F, GI-R (8 h) group; G, GI-R (16 h) group; H, LHA+GI-R+0.5 μg Baclofen group; I, LHA+GI-R+1.5 μg Baclofen group; J, LHA+GI-R+3.0 μg. *n* = 6.

At first, we sought to determine whether BABA_B_ receptor agonist Baclofen (0.5, 1.5, 3.0 μg) exerts its protective effect on GI-R injury. We found that Baclofen dose-dependently attenuated GI-R injury (Figure 1, H–J). Furthermore, the gastric mucosal injury index (GMII) was time-dependently changes and the GMII peaked in the reperfusion 1 h (Figure 1, A–G).

### Chemical stimulation of the FN or microinjection of baclofen or CGP35348 into the LHA in GI-R injury rats

**Figure 2:**
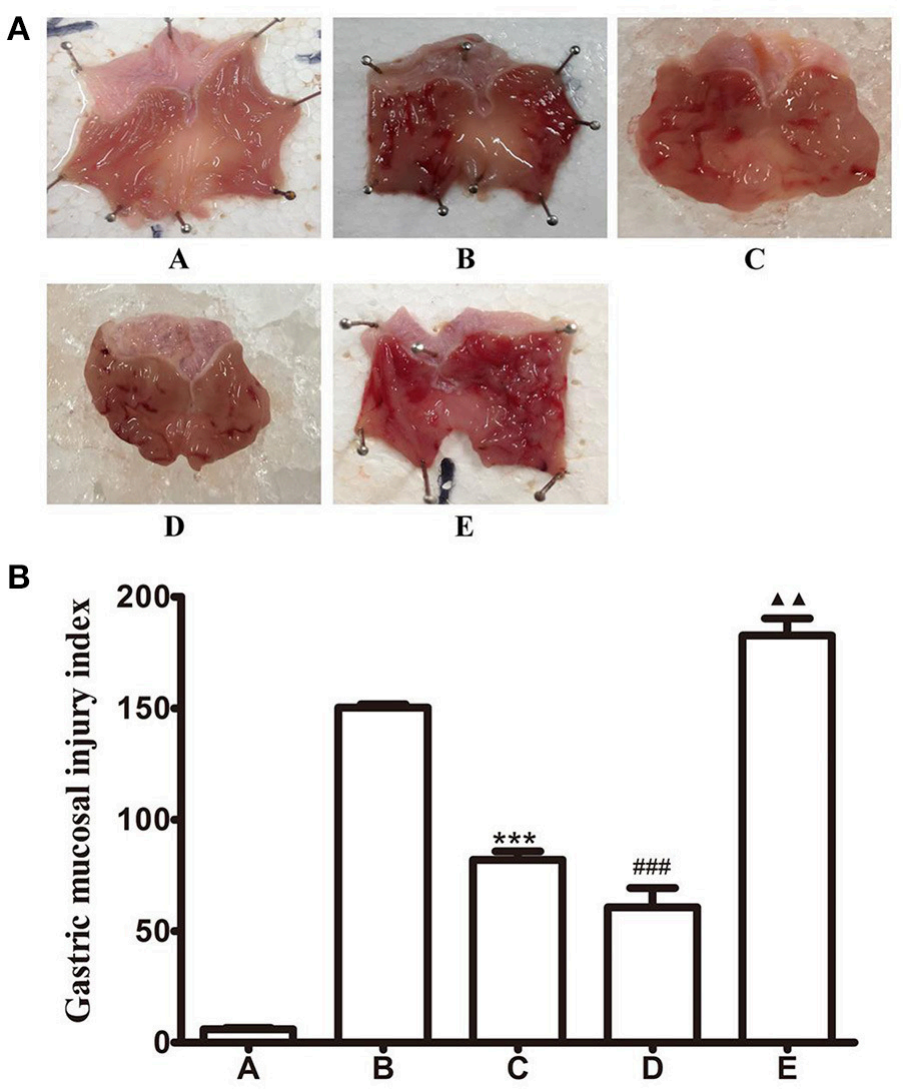
Effects of FN stimiulation, GABA_B_ receptor agonist or antagonist microinjected into LHA on GI-R injury. **(A)** Representative macroscopic photograph of gastric tissue in different groups; **(B)** Effects of chemical stimulation of FN or microinjection of GABA_B_ receptor agonist or antagonist into LHA on GI-R injury in rats. ^***^P < 0.001, as compared with the GI-R group; ^###^*P* < 0.001, as compared with the GI-R group; ^▴▴^*P* < 0.01, as compared with the GI-R group. A, Normal group; B, GI-R group; C, FN+GI-R+*L*-Glu group; D, LHA+GI-R+Baclofen group; E, LHA+GI-R+CGP35348 group. *n* = 5.

As our previous reports, stimulation of FN with *L-*Glu attenuated the gastric ischemia-reperfusion injury in rats. Here, we want to investigate the effects of GABA_B_ receptor in LHA on GI-R injury. Microinjection of baclofen, a GABA_B_ receptor agonist or antagonist CGP35348 into the LHA. We found that unilateral microinjection of GABA_B_ receptor agonist baclofen into the LHA attenuated GI-R injury. However, microinjection of GABA_B_ receptor antagonist CGP38348 aggravated the GI-R injury (Figure 2). This result indicates that GABA_B_R in the LHA plays a critical role in protecting the GI-R injury.

### GABA_B_R expression and cellar location were detected by immunofluorescence

**Figure 3:**
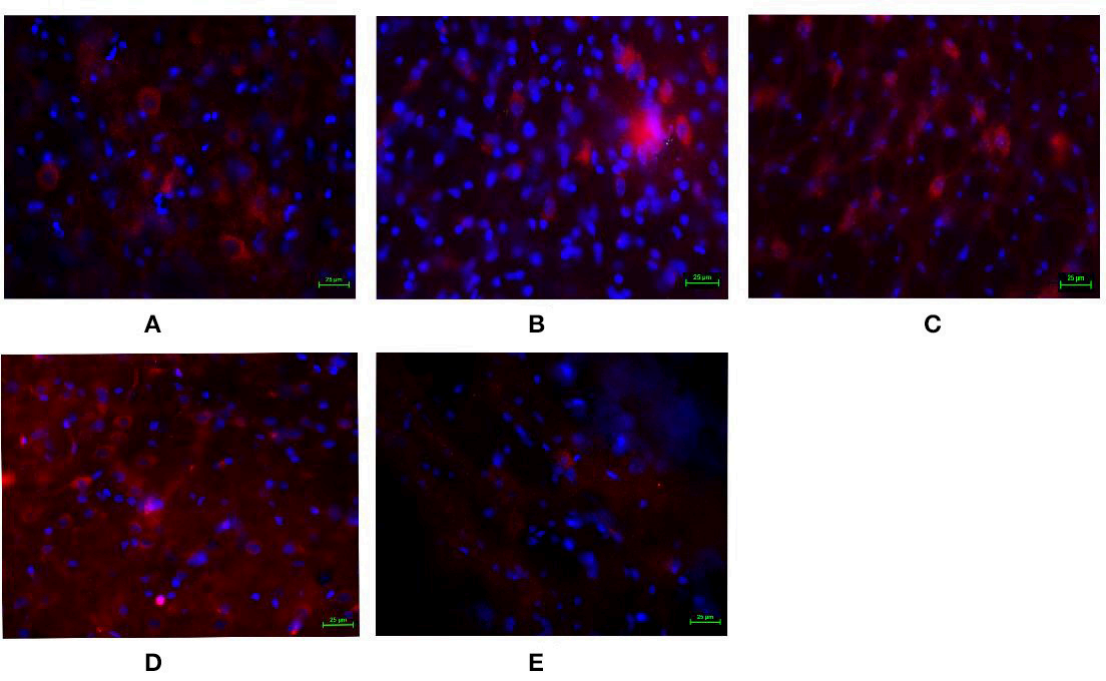
Immunofluorescence expression of GABA_B_ receptor in the LHA. Immunofluorescence analysis of expression of GABA_B_ receptor in the LHA. **(A)** Normal group; **(B)** GI-R group; **(C)** FN+GI-R+*L*-Glu group; **(D)** LHA+GI-R+ Baclofen group; **(E)** LHA+GI-R+CGP35348 group. *n* = 5.

In order to detect whether the GABA_B_R expressed in the LHA and to furtherly investigate the effects of baclofen and CGP35348 on GABA_B_R expression, immunofluorescence technology was adopted to detect the GABA_B_ receptor. As shown in Figure 3, compared with the GI-R group, microinjection of *L*-Glu into the FN or GABA_B_R agonist baclofen into the LHA up-regulated the GABA_B_R expression. However, microinjection GABA_B_R antagonist CGP35348 into the LHA down-regulated the GABA_B_R expression.

### Expression of GABA_B_R and *P*-GABA_B_R in the LHA

**Figure 4:**
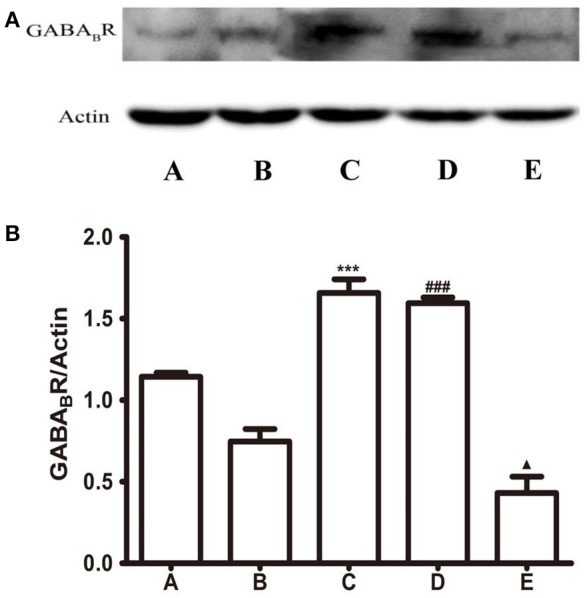
Protein expression of GABA_B_ receptor in the LHA. **(A)** Western blot analysis of expression of GABA_B_ receptor. **(B)** Quantitative analysis of GABA_B_ receptor expression in the LHA. β-Action was used as a loading control. ^***^P < 0.001, as compared with the GI-R group; ^###^*P* < 0.001, as compared with the GI-R group; ^▴^*P* < 0.05, as compared with the GI-R group. A, Normal group; B, GI-R group; C, FN+GI-R+*L*-Glu group; D, LHA+GI-R+Baclofen group; E, LHA+GI-R+CGP35348 group. *n* = 5.

Next, the expression of GABA_B_R in the LHA was detected by western blot assay. As Figure 4 showed, microinjection of *L-*Glu into the FN or baclofen into the LHA significantly increased the expression of GABA_B_R in the LHA, but microinjection CGP35348 decreased the expression of GABA_B_R.

**Figure 5:**
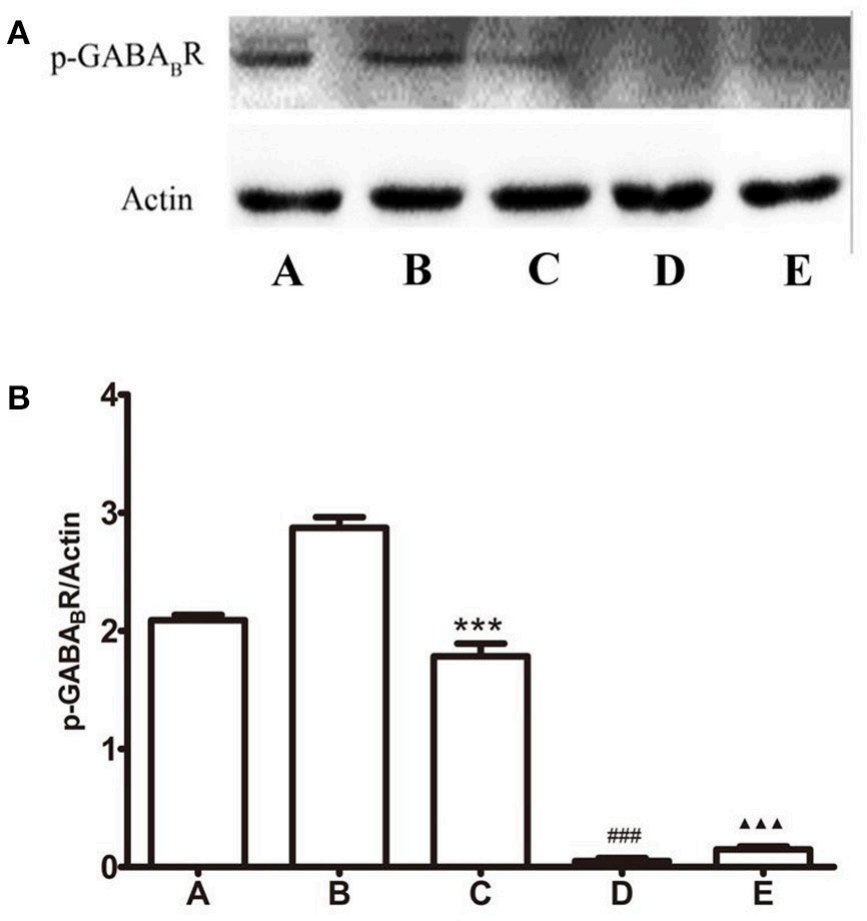
Protein expression of *P*-GABA_B_ receptor in the LHA. **(A)** Western blot analysis of expression of *P*-GABA_B_ receptor. **(B)** Quantitative analysis of *P*-GABA_B_ receptor expression in the LHA. β-action was used as a loading control. ^***^P < 0.001, as compared with the GI-R group; ^###^*P* < 0.001, as compared with the GI-R group; ^▴▴▴^*P* < 0.001, as compared with the GI-R group. A, Normal group; B, GI-R group; C, FN+GI-R+*L*-Glu group; D, LHA+GI-R+ Baclofen group; E, LHA+GI-R+CGP35348 group. *n* = 5.

*P*-GABA_B_R was detected by western blot assay. As shown in Figure 5, compared with the GI-R group, *L-*Glu into the FN or baclofen into the LHA lead the *P*-GABA_B_R significantly decreased, *P* < 0.001. The level of *P*-GABA_B_R is also quite low in the microinjection CGP35348 group, this may be related with the lower expression of GABA_B_R. The expression levels of *P*-GABA_B_R was opposed with GABA_B_R.

### The expression of GABA_B_R-mRNA in GI-R injury rats

**Figure 6:**
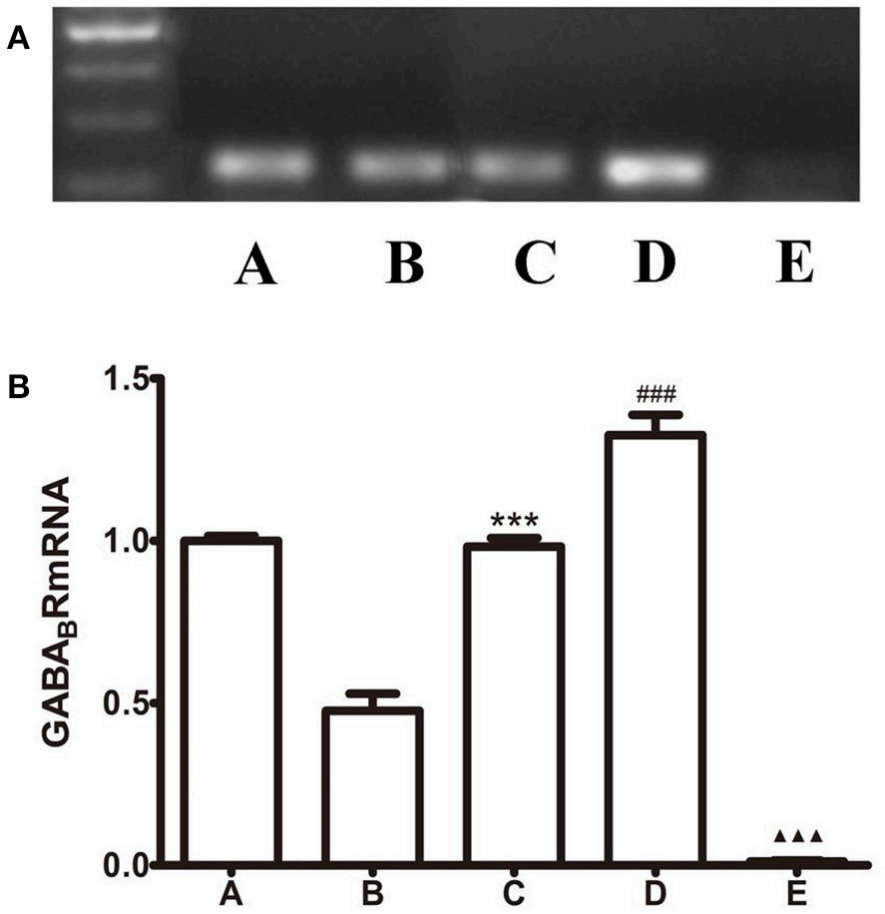
Effects of FN stimulation, GABA_B_ receptor agonist or antagonist microinjected into LHA on GABA_B_R-mRNA expression. **(A)** PCR analysis of expression of GABA_B_ receptor. **(B)** Quantitative analysis of GABA_B_ receptor expression in the LHA. β-action was used as a loading control. ^***^P < 0.001, as compared with the GI-R group; ^###^*P* < 0.001, as compared with the GI-R group; ^▴▴▴^*P* < 0.001, as compared with the GI-R group. A, Normal group; B, GI-R group; C, FN+GI-R+*L*-Glu group; D, LHA+GI-R+ Baclofen group; E, LHA+GI-R+CGP35348 group. *n* = 5.

To visualize the expression of GABA_B_R in the LHA, we perform PCR and quantitative real-time PCR experiments. As shown in Figure 6, the amplification of GABA_B_ receptors from rats' brain cDNA revealed that all transcripts present in the LHA. Relative abundance of GABA_B_ receptors in the normal group was deviated from others and reached a peak in the Baclofen treatment group. But CGP35348 treatment inhibited the transcript. In addition, microinjection of *L*-Glu into the FN or baclofen into the LHA increase the GABA_B_R-mRNA expression, compared with the GI-R group. The levels of GABA_B_R-mRNA were identified with the levels of GABA_B_R protein in each corresponding groups.

### Expression levels of NOX2 and NOX4 in the LHA

*L-*Glu microinjected into the FN or baclofen microinjected into the LHA attenuated the GI-R injury, but CGP35348 microinjection into the LHA aggravate the GI-R injury. Meanwhile, as shown in Figures 4, 5, *L-*Glu microinjected into the FN or baclofen microinjected into the LHA enhanced the expression levels of GABA_B_R protein or the GABA_B_R-mRNA. According to these results, we want to investigated how the baclofen or the CGP35348 influences gastric ischemia-reperfusion injury, and whether baclofen or CGP35348 affect the NOX2/NOX4 expression and ROS production?

**Figure 7:**
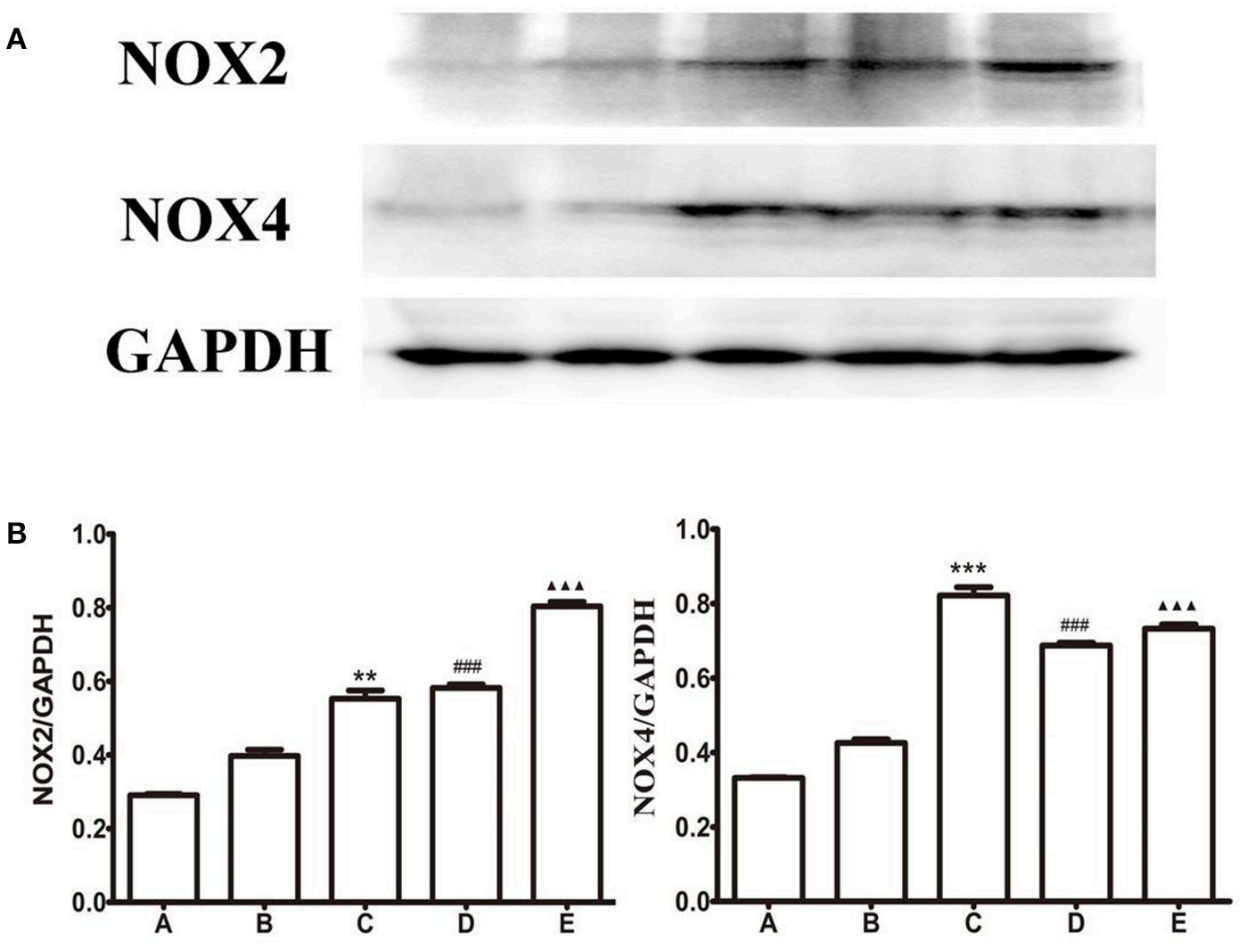
Protein expression of NOX2 and NOX4 in the LHA. **(A)** Western blots of NOX2, NOX4, and GAPDH in LHA. **(B)** Quantitative analysis of NOX2, NOX4 expression in the LHA. GAPDH was used as a loading control. ^***^P < 0.001 (^**^P < 0.01), as compared with the GI-R group; ^###^*P* < 0.001, as compared with the GI-R group; ^▴▴▴^*P* < 0.001, as compared with the GI-R group. A, Normal group; B, GI-R group; C, FN+GI-R+*L*-Glu group; D, LHA+GI-R+ Baclofen group; E, LHA+GI-R+CGP35348 group. *n* = 5.

As shown in Figure 7, compared with the GI-R group, the expression of NOX2 and NOX4 were all up-regulated in different drugs treatment groups. The gastric ischemia-reperfusion induces up-regulation of NOX2 and NOX4, baclofen or CGP35348 treatment doesn't reverse this changes. This result indicates that the protection of microinjection of GABA_B_R agonist in the LHA in GI-R injury isn't mediated via oxidative stress pathway.

**Figure 8:**
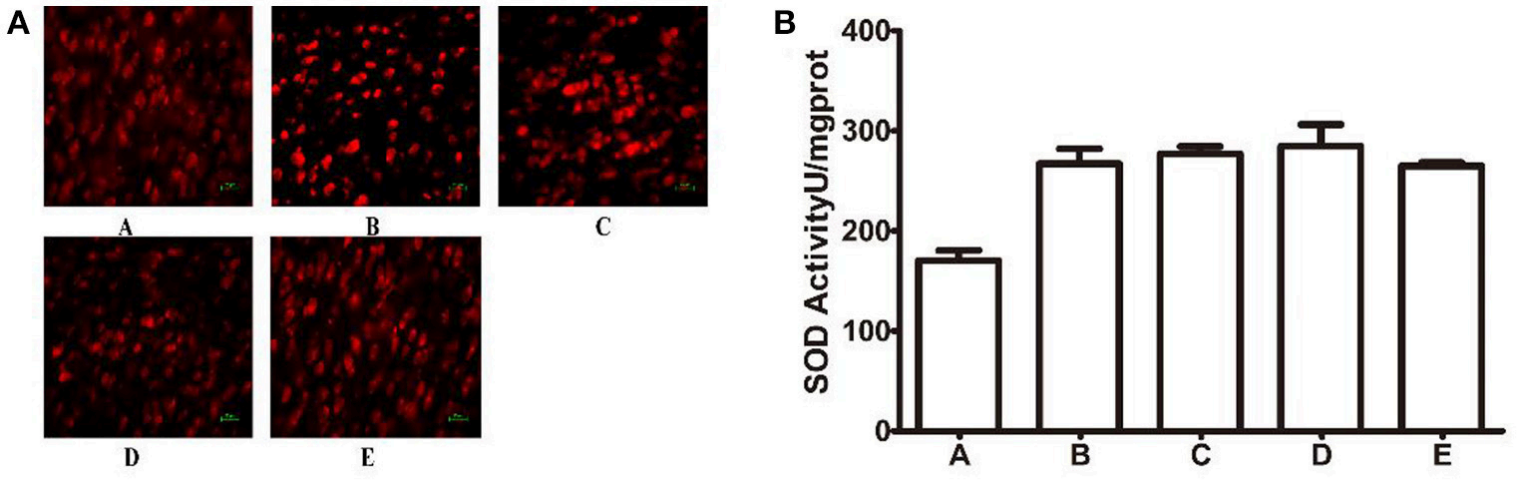
Changes of ROS generation in different groups. **(A)** ROS Fluorescent Probe-DHE; **(B)** SOD. A, Normal group; B, GI-R group; C, FN+GI-R+*L*-Glu group; D, LHA+GI-R+ Baclofen group; E, LHA+GI-R+CGP35348 group. *n* = 5.

Total SOD activity was considered as an indication of the anti-oxidative properties of cells. The ROS fluorescent Probe-DHE was detected on the frozen section. As shown in Figure 8A, compared to the GI-R group, there was no change in different drugs treatment groups. Compared to the GI-R group, SOD activity in the LHA had little significantly change in the other groups, as shown in Figure 8B.

### Expression of IL-1β, NOX2, and NOX4 in the gastric mucosa

**Figure 9:**
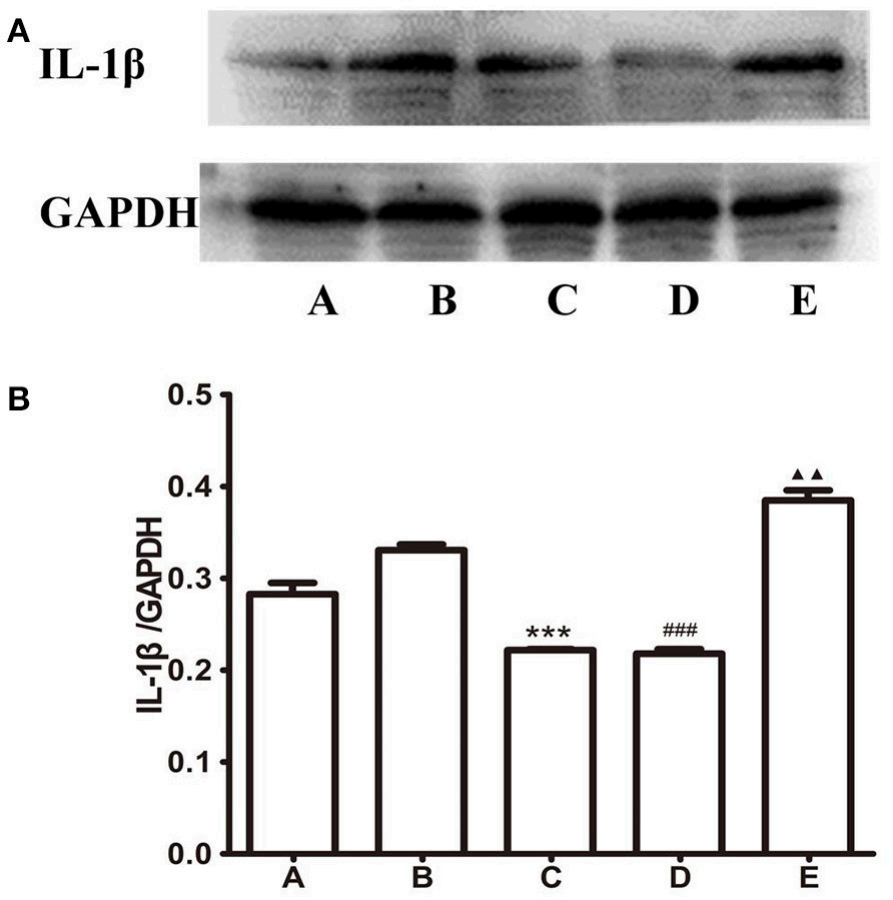
Expression of IL-1beta in the gastric mucosa. **(A)** Western blots of IL-1β and GAPDH in gastric mucosa. **(B)** Quantitative analysis of IL-1β expression in the gastric mucosa. GAPDH was used as a loading control. ^***^P < 0.001, as compared with the GI-R group; ^###^*P* < 0.001, as compared with the GI-R group; ^▴▴^*P* < 0.01, as compared with the GI-R group. A, Normal group; B, GI-R group; C, FN+GI-R+*L*-Glu group; D, LHA+GI-R+ Baclofen group; E, LHA+GI-R+CGP35348 group. *n* = 5.

The above studies indicated that the protective effect of Baclofen was not mediated by the oxidative stress pathway in brain, then we furtherly investigated the relations between GABA_B_ receptor and gastric mucosa injury. We detected IL-1β, NOX2, and NOX4 in the gastric mucosa. As shown in Figure 9, compared to B group, the expression of IL-1β decreased in *L*-Glu treatment group, ^***^P < 0.001 and D group, ^###^*P* < 0.001; the expression level of IL-1β increased in E group, ^▴▴^*P* < 0.01.

**Figure 10:**
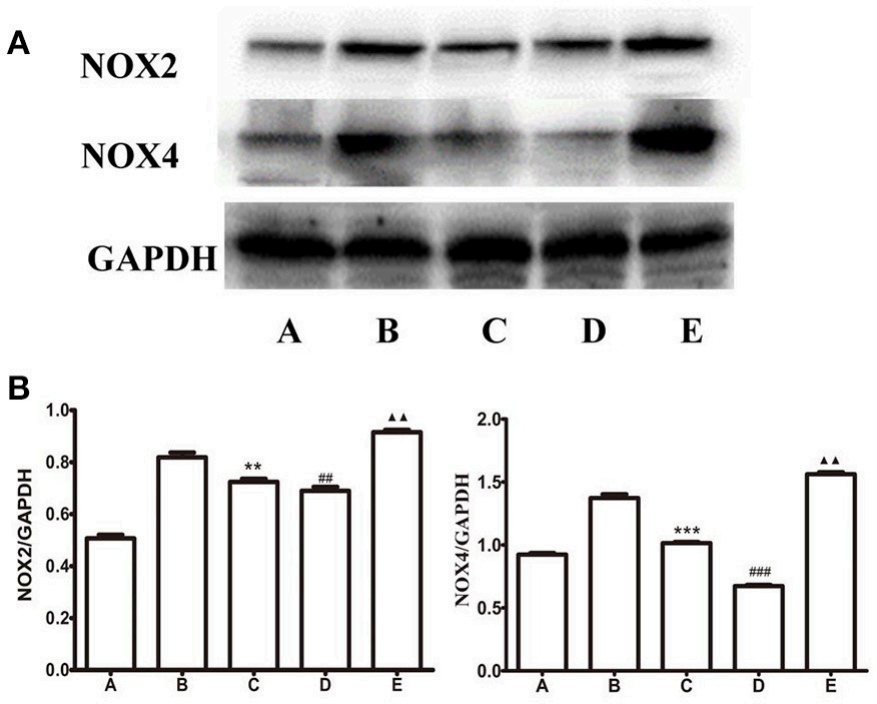
Expression of NOX2 and NOX4 in the gastric mucosa. **(A)** Western blots of NOX2, NOX4, and GAPDH in gastric mucosa. **(B)** Quantitative analysis of NOX2, NOX4 expression in the gastric mucosa. GAPDH was used as a loading control. *n* = 5, ^**^P < 0.01 (^***^P < 0.001), as compared with the GI-R group; ^##^*P* < 0.01 (^###^*P* < 0.001), as compared with the GI-R group; ^▴▴^*P* < 0.01, as compared with the GI-R group. A, Normal group; B, GI-R group; C, FN+GI-R+*L*-Glu group; D, LHA+GI-R+ Baclofen group; E, LHA+GI-R+CGP35348 group.

Compared to B group, the expression level of NOX2 decreased in C group, ^**^P < 0.001 and D group, ^##^*P* < 0.001; the expression level of NOX2 increased in E group, ^▴▴^*P* < 0.01, as shown in Figure 10. Compared to B group, the expression of NOX4 decreased in C group, ^***^P < 0.001 and D group, ^###^*P* < 0.001; the expression level of NOX4 increased in E group, ^▴▴^*P* < 0.01, as shown in Figure 10.

### Expression of CGRP in the gastric mucosa

**Figure 11:**
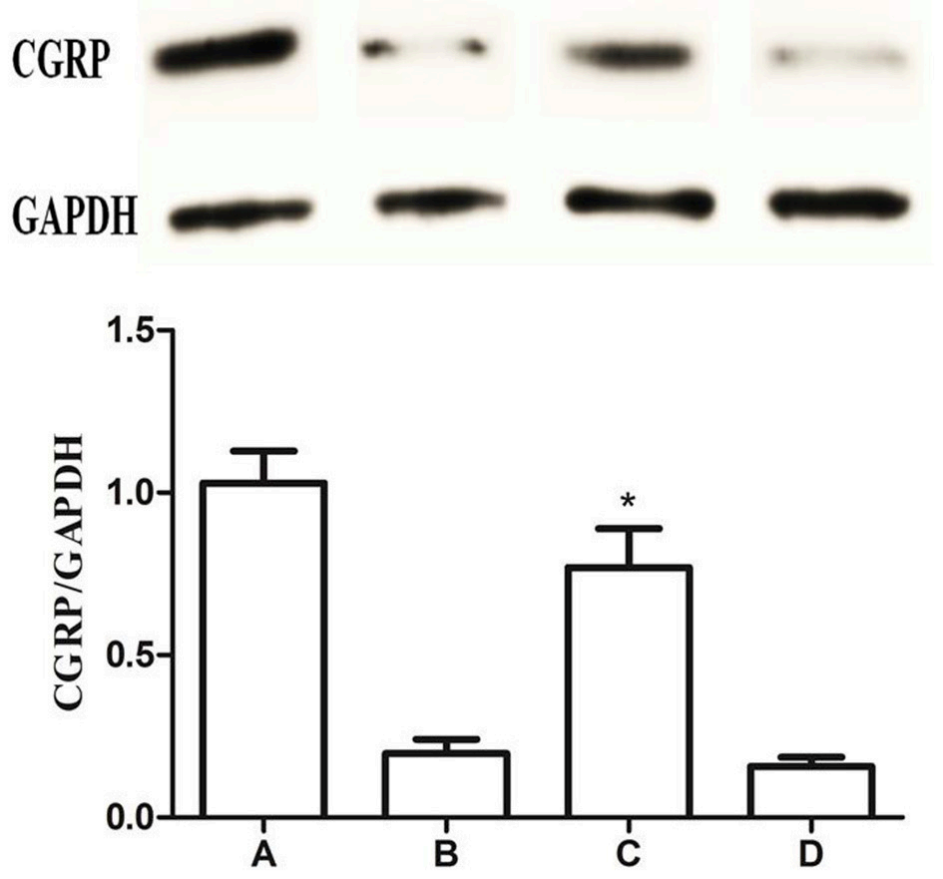
Expression of CGRP in the gastric mucosa. Western blots of CGRP and GAPDH in gastric mucosa. Quantitative analysis of CGRP expression in the gastric mucosa. GAPDH was used as a loading control. ^*^P < 0.001, as compared with the GI-R group. A, Normal group; B, GI-R group; C, LHA+GI-R+Baclofen group; D, LHA+GI-R+CGP35348 group. *n* = 5.

Base on our previous report that CGRP could attenuate the GI-R injury, we determined whether Baclofen in the LHA can induce the release of CGRP in the gastric tissue. As shown in Figure 11, compared with the GI-R group, the expression level of CGRP increased LHA+GI-R+Baclofen group, ^*^P < 0.01.

### Expression of NOX2 and NOX4 in the gastric mucosa after CGRP (iv)

**Figure 12:**
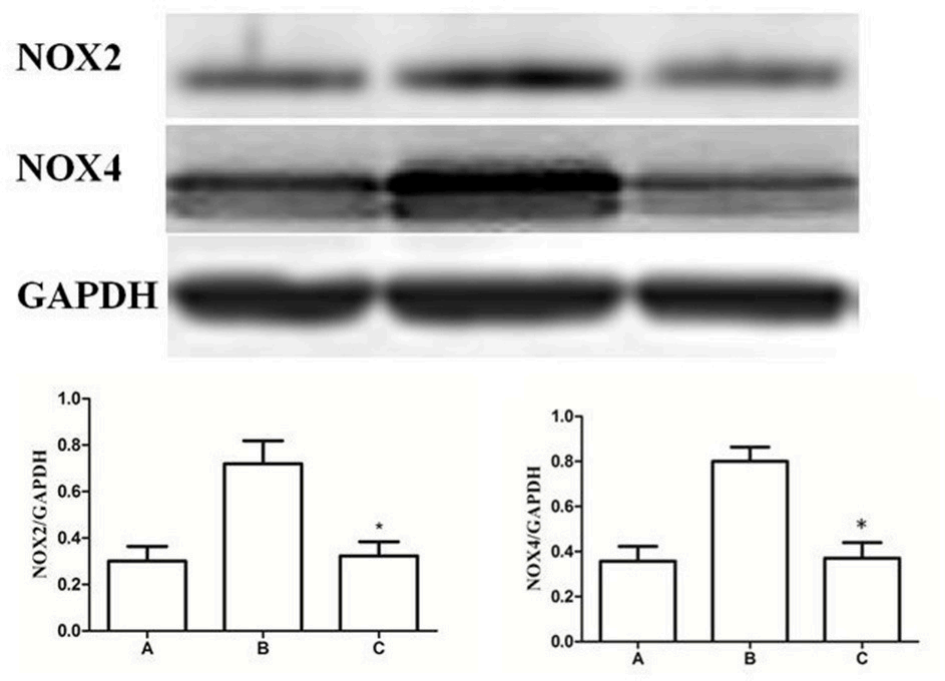
Expression of NOX2 and NOX4 in the gastric mucosa. Western blots of NOX2, NOX4, and GAPDH in gastric mucosa. Quantitative analysis of NOX2 and NOX4 expression in the gastric mucosa. GAPDH was used as a loading control. ^*^P < 0.001, as compared with the GI-R group. A, Normal group; B, GI-R group; C, LHA+GI-R+CGRP group. *n* = 5.

Finally, to confirm the CGRP attenuated the GI-R injury via anti-oxidant pathway in gastric mucosa, CGRP was administrated (iv) and levels of superoxide anion radical generating enzymes NOX2/4 were measured by immunoblot. As shown in Figure 12, compared with the GI-R group, CGRP attenuated the expression of NOX2 and NOX4, ^*^P < 0.001.

## Discussion

The present study was designed to determine the effects of GABA_B_ receptor of brain on gastric ischemia-reperfusion-induced injury. Firstly, we identified that GABA_B_ receptor was expressed in the LHA by immunofluorescence. The GABA_B_ receptor was distributed throughout the LHA just like GABA_A_ receptor.

Next, in order to investigate how does the GABA_B_ receptor in the LHA affect the GI-R injury. GABA_B_ receptor agonist baclofen microinjected into the LHA attenuate the GI-R induced injury. But a similar microinjection of CGP35348 (GABA_B_ receptor antagonist) aggravated the GI-R injury. According to the western blot analysis in the LHA, microinjection of GABA_B_ receptor agonist into the LHA up-regulate the GABA_B_ receptor expression, but microinjection of CGP35348 attenuates the GABA_B_ receptor expression. These results indicated that the GABA_B_ receptor overexpression in the LHA protect against the GI-R injury in rats.

The previous studies demonstrated that GBAB_A_R in the LHA was actively involved in the pathophysiology of GI-R injury (Zhu J. Z. et al., 2012; Zhu S. P. et al., 2012). And a recent report provided that GABA_A_R overexpression in the LHA resulted in a marked protection against GI-R injury (Gao et al., 2015). Our study demonstrated that GABA_B_ receptor affect the GI-R injury has similar effects with the GABA_A_R. Previous studies were focused on testing the physiological indexes like mucosal blood flow, the mucosal cell proliferation and apoptosis after microinjection of the GABA_A_R agonist. In our experiment, the protein and mRNA expression of GABA_B_R, *P*-GABA_B_R, oxidative stress signal indicators were used to prove the protective effects of microinjection the GABA_B_R agonist into LHA on gastric ischemia-reperfusion injury in rats. Our results indicated that the protective effects of GABA_B_ receptor on GI-R injury might be mediated via up-regulating the GABA_B_ receptor protein and GABA_B_R-mRNA expression in the LHA. Microinjection of the GABA_B_R agonist increased the GABA_B_R expression and decreased the *P*-GABA_B_R expression.

Numerous studies have suggested that excessive production of ROS or oxygen free radicals could be important initiating factors or pathogenic factors mediating GI-R injury (Itoh and Guth, 1985; Ishii et al., 2000). According to previous studies, we want to investigated whether the GABA_B_ receptor protect the GI-R against the injury through the anti-oxidative stress pathway. And then the expression of NADPH oxidase and the production of ROS were measured. One important finding in this study is that the protective effects of GABA_B_ receptor wasn't mediated by anti-oxidant pathway in the brain. In other organs, the oxygen free radicals play an important role in the development of I/R injury, but the NOX2, NOX4, SOD, and ROS have not been obviously changed in our experiments.

IL-1β is a member of the interleukin 1 family of cytokines, which is produced by activated macrophages. And this cytokine is an important mediator of the inflammatory response, and is involved in cell apoptosis. The expression of IL-1β, NOX2, and NOX4 in the gastric mucosa was detected and the results showed that pretreatment of baclofen microinjected into LHA decreased the expressions of IL-1β, NOX2, and NOX4. In reverse, pretreatment of CGP35348 microinjected into LHA increased the expressions of IL-1β, NOX2, and NOX4 in the gastric mucosa. Our data enabled us to ascertain which of the added drugs targeted the GABA receptor in the LHA and acted through NOX2/NOX4 signaling. Base on our previous study demonstrated that CGRP released form dorsal root ganglion (DRG) could attenuate the GI-R injury. Therefore, firstly, we detected the expression of CGRP in gastic mucosa in the induction of GABA_B_ Receptor agonist; Secondly, the NOX2/NOX4 in gastric mucosa were detected in the induction of CGRP. And our results showed that baclofen in the LHA induced an increase of expression of CGRP, CGRP (iv) attenuated the expression of NOX2/NOX4.

**Figure 13:**
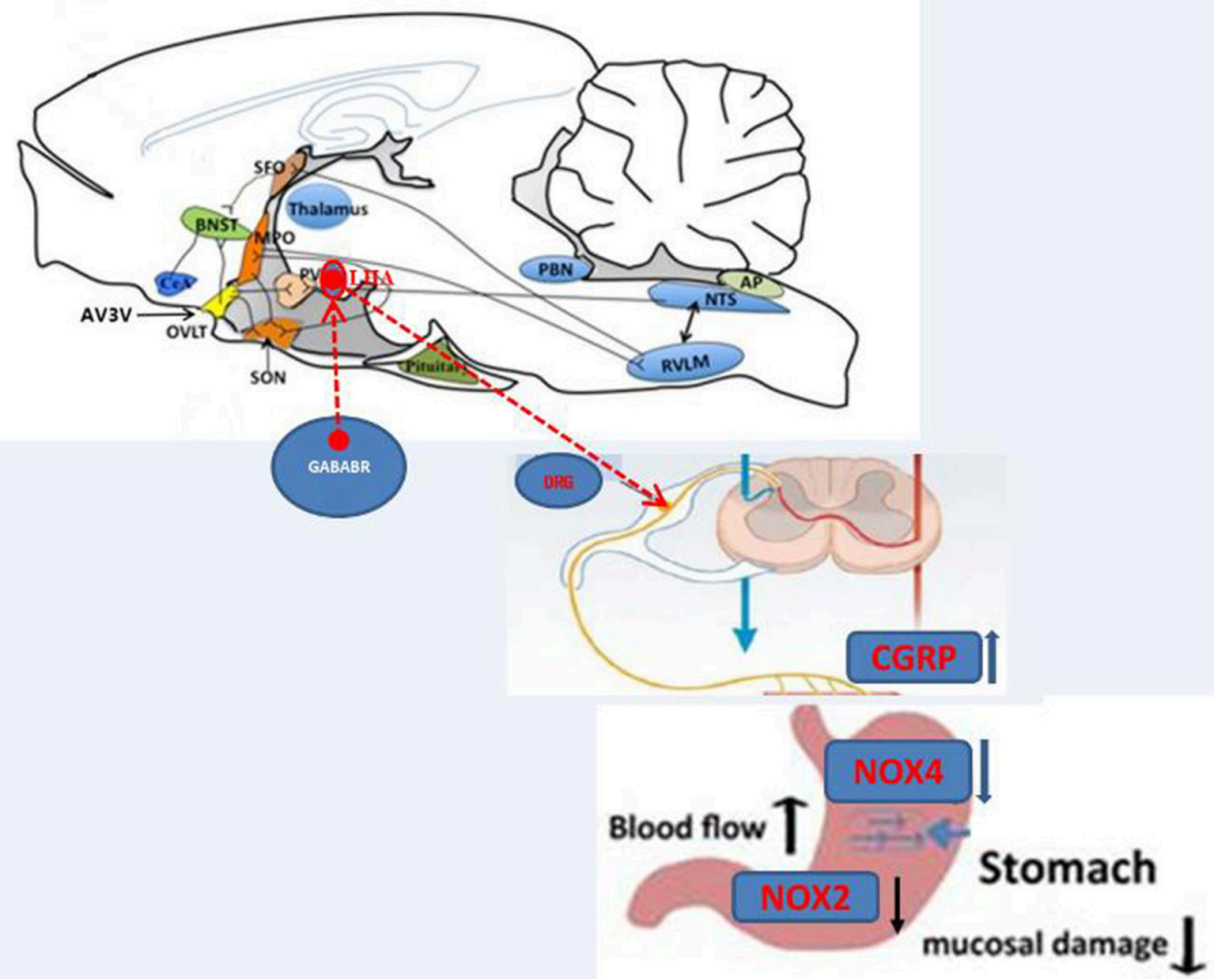
Illustration of the effects of GABA_B_R in the LHA on GI-R injury.

In conclusion, the GABA_B_ receptor in the LHA attenuates GI-R injury by neuroendocrine and intracellular signal transduction pathway. As shown in Figure 13, the protective effects of GABA_B_ receptor may be mediated by peripheral neuron pathway, and the possible regulative mechanism is that the activation of GABA_B_ receptor (Balcofen) trigger neurons to release neurotransimitter CGRP, which improved the gastric mucosal blood flow. Then the CGPR attenuated GI-R injury via anti-oxidant pathway (decrease of NOX2/NOX4) in the gastric mucosa.

## Conclusion

The protection of GABA_B_ receptor in the LHA against GI-R injury could be related to the decrease of IL-1β, NOX2, and NOX4 in the gastric mucosa.

## Author contributions

Design of the study and analyzed the data: DD, DC, ZD, and YL with the inputs from all authors. Write the paper: HZ, LG, and TZ with the inputs from all authors. Perform the experiment and analysis the data: HZ, LG, LH, ZL, HH, and FC.

### Conflict of interest statement

The authors declare that the research was conducted in the absence of any commercial or financial relationships that could be construed as a potential conflict of interest.
